# Mapping social capital across Wales (UK) using secondary data and spatial analysis

**DOI:** 10.1007/s43545-023-00639-1

**Published:** 2023-03-06

**Authors:** Muhammad Irfan, Kelly Buckley, Sin Yi Cheung, James J. Lewis, Aleksandra Koj, Hywel Thomas

**Affiliations:** 1 Y Lab – the Public Services Innovation Lab for Wales, School of Social Sciences, Cardiff University, Cardiff, UK; 2 School of Social Sciences, Cardiff University, Cardiff, UK; 3 School of Engineering, Cardiff University, Cardiff, UK

**Keywords:** Social capital, Social capital mapping, Spatial social capital

## Abstract

Social capital, a powerful community resource based on trust, relationships, norms, culture, values, networks and belonging, could shape the acceptance, cooperation, and involvement of citizens towards new policies or interventions. In past, connections of social capital have been studied in relation to human health, wellbeing, social and economic development. More recently, social capital has been studied with respect to human resilience and adaptation to climate change. We argue that social capital could also play a vital role in our efforts to reduce carbon footprint through behaviour change, a shift on shared local renewable energy resources, and adoption of low carbon technologies. In Wales (UK) there is no national scale dataset, reflecting its social capital landscape, that could be used for designing the right policies/interventions in this context, based on an expected level of trust, cooperation, and support within the communities. This paper is an effort to fill this data gap using secondary datasets. Firstly, a literature review is carried out to identify the indicators of social capital (cognitive and participatory). Secondary datasets have then been identified and acquired. Geospatial analysis has been carried out to produce the criterion maps for various indicators of social capital. Finally, Analytical Hierarchy Process is applied to generate a social capital map of Wales combining these indicators together. For validation of the produced data, social capital’s known correlations were tested with crime rates, income level and multiple deprivations.

**Supplementary Information:**

The online version contains supplementary material available at 10.1007/s43545-023-00639-1.

## Introduction

### Background

In 2019, Wales became the first parliament in the world to declare a climate emergency and has recently committed to become Net Zero by 2050 (Carbon Budget-2 2021). Wales commitments on carbon reduction are mostly on the demand side. Therefore, in the Welsh context, a significant reduction in demand through increased efficiency and behaviour change in our society will be key to decarbonisation (CarbonBudget-2 2021).

These ambitious net zero targets bring unique challenges and opportunities. Furthermore, it raises questions as to how Welsh communities can gain maximum environmental and socio-economic co-benefits from net zero initiatives.

Literature suggests that communities with higher social capital are more likely to have climate-mitigation behavioural intention (Hao et al. 2020). They are also more likely to show greater support for climate policy (Hao et al. 2020). In other contexts, we know that there is a link between social capital and environmental action and literature suggests that vibrant communities have inherent capacities to work collectively and better adapt to climate change (see for example Pretty and Ward 2001, Adger 2010, Liu et al. 2014, Petzold and Ratter 2015, Tang 2016).

A shared vision among residents in the community is crucial to bring people onboard in driving any behavioural change. Behaviour change is one of the key barriers to the successful uptake and implementation of decarbonisation interventions: as argued by Whitmarsh et al. (2021) many behavioural models are reductive and neglect the need to focus on high-emitting behaviours and high-emitting groups.

Above all, collective action at the local level and participation of civil society is also essential, which is where social capital could be a potentially powerful resource that can be mobilised to achieve a common goal or a collective good. Social capital is a multidimensional and complex concept, which has gained traction in recent years in research around community and community wellbeing (Dodds 2016). Although several conceptualisations of social capital exist, the three main ones often cited are attributable to Bourdieu, Coleman and Putnam (Daykin et al. 2021). Based largely on the premise that social relationships affect social processes, it has been used in several fields to understand key processes that affect outcomes, such as in public health research (Gregson et al. 2004; Villalonga-Olives et al. 2018; Saville and Thomas 2022). Bourdieu’s work connects social capital to economic and cultural capital, making it a concept that helps to critically explore how power operates, specifically in relation to networks (Swartz 2012). However, Putnam’s (1993) conceptualisation of the term sees social capital as a positive resource comprising trust, norms, and networks that, through these bonds, can facilitate social cohesion. In this conceptualisation, social capital consists of social networks and the norms of reciprocity and trustworthiness that arise from them (Putnam 1993). It is the institutions, relationships, attitudes, and values that govern interactions among people and contribute to economic and social development (Grootaert and Van Bastelaer 2002). Therefore, Putnam’s (1993) notions of ‘bonding and bridging’ capital, with a focus on belonging and shared identity, makes this definition of social capital an appropriate conceptualisation through which we can understand the acceptability and take-up of decarbonisation initiatives.

### Knowledge gap and justification of research

Knowing this spatially and socially differentiated connection exists between communities and their ability to adapt to climate change, we think that the same can be utilised in shaping public perceptions about new initiatives (such as community energy schemes and smart local energy systems) to accelerate decarbonisation and help reduce our carbon emissions.

However, there is currently a data and knowledge gap in Wales to reflect the spatial variations in social capital level across the country. We believe that a national scale map showing the geographical variations of Social Capital level can be useful for the decision makers in national and local governments to design relevant policies and location-specific carbon reduction interventions considering its level of social capital. For example, a community renewable energy schemes requires a higher level of cooperation among people for the scheme to be successful. Fraser (2021) analysed that higher social capital generally affect the solar projects siting in a positive way, however it varies geographically. Similarly, Su et al. (2023) assessed the links between diverse energy consumption structure and social capital in the southern Shaanxi province of China. The study results show that high social capital has a significant positive impact on the use of solar energy (Su et al. 2023).

Summarising this evidence found in the literature, we can state that the areas with higher social capital (bonding, bridging, and linking) have a general tendency to adopt to local renewable energy resources and are more prone to behaviour change to reduce their carbon footprint. If this concept is considered in the net zero policies and planning, decarbonisation could be accelerated by targeting areas with higher social capital. However, this doesn’t imply that areas with relatively lower social capital should be ignored. Rather specialised interventions should be designed for such areas so that a just transition is achieved and people/communities with low social capital also get co-benefits of such initiatives. Also, by designing the right interventions in the light of social capital of an area can help avoid social problems potentially arising after setting off the projects, for example those highlighted by Few et al. (2007) and Anderson et al. (2012).

### Key indicators of social capital

A literature review has been carried out to identify key indicators of social capital, drawing on both its objective and subjective aspects. The objective aspects refer to the observable and tangible networks, associations, and institutions in a society including civic participation. The non-tangible (subjective) aspects relate to issues such as mutual trust, reciprocity, and generally accepted attitudes, norms, and behaviours (Putnam 1993; Grootaert and Van Bastelaer 2002; Foxton and Jones 2011). Using these defining characteristics, a general assessment of social capital can be mapped across a geographical region. For example, crime rate, voter turnout and vote share for the winning party have been employed as proxies for measuring social capital in South Africa to understand the disparities of a renewable energy initiative in different geographical areas (Fraser 2021). Voter turnout is a key indicator of civic participation since it reflects involvement in local and national affairs, and perceptions of ability to influence them (Foxton and Jones 2011). How people feel about their neighbourhood can shape local areas and can affect people’s behaviour, and in turn people can become more co-operative and tolerant (Foxton and Jones 2011).

Ahn and Davis (2020) used mixed methods approach to analyse sense of belonging as a measure of social capital. The results provide strong evidence that sense of belongingness and social capital are theoretically and empirically connected, and this connection can be clearly traced and measured (Ahn and Davis 2020).

Many examples in the literature support the argument that higher social capital results in lower criminal victimisation and therefore crime rates have been used frequently to assess the level of social capital in an area (see for example Akçomak and Ter Weel 2012a; Roh and Lee 2013).

Targeted surveys and demographic information has also been used in order to measure social capital, e.g. the Scottish Government has examined social capital across Scotland using questions asked in the Scottish Household Survey 2018 (Kerri McClymont 2020). These questions were grouped in four categories: (i) Social Network (frequency of socialising, helping neighbours and relying on them for advice and help); (ii) Social Participation (volunteer work); (iii) Community Cohesion (views about neighbourhood including belongingness, safety, trust, and reciprocity); and iv) Community Empowerment (influencing local decisions to improve their neighbourhood) (Kerri McClymont 2020). Based on this data analysis, a report has been published which explains how capital is distributed across social groups and geographies (including local authority areas, areas of higher deprivation, and urban and rural areas), also highlighting which of these groups and places have relatively lower levels of social capital.

Based on the literature review presented here, we identified 5 key variables that can be used to measure a general relative measure of social capital for an area. Table 1 shows these variables, which category of social capital they fall into, and what other data can be used as a proxy for measuring social capital in a geographic context.

**Table 1:** Key indicators for measuring social capital

Indicators identified for measuring social capital		
Bridging social capital	Civic participation	Voter turnout as an indicator of civic participation and engagement and has frequently been used as a measure of bridging social capital. Voter turnout is also often considered as an indicator of linking social capital
	Social participation	Frequency and time spend by people in religious services, joint action local groups and unpaid volunteer work is a measure of social participation has been frequently used in measuring bridging social capital
Bonding social capital	Views of the local area	People’s views about their local area reflect their belongingness to the local area and has been used frequently as a measure of bonding social capital. Views can cover a range of different subjects for example how safe people feel at home, walking or cycling in their neighbourhood etc
	Reciprocity and trust	Another reflection of bonding social capital is how much people trust their neighbours, family and friends. How people treat each other in an area and if people from different backgrounds get on well with each other in their localities. One indication of trust in the neighbourhood is that people feel safe for their children to play outside
Other correlations	Crime	Low crime rates are used frequently as a measure of higher social capital. For this indicator, rates of recorded crime standardised by population size for a given area can be used as direct measurement. Because it is a direct measurement, it can also be used for the validation of social capital measurements from above indicators
	Income	Higher income level of communities is repeatedly used as a correlation of higher social capital. Because it is a direct measure, It can also be used for the validation of social capital measured through other indicators that define bonding and bridging social capital

### Secondary datasets used for social capital mapping

Other proxy variables have been commonly used in research across many fields (from public health to environmental and public policy) to measure dimensions of social capital.

The most used proxy variables are crime rates and income levels. A review of relevant literature suggests that high levels of social capital results in positive outcomes on the local socio-economic context: for example, higher social capital should theoretically contribute to the better standard of living (Putnam 1993; Gregson et al. 2004; Anderson et al. 2012; Liu et al. 2014; Fernández Aldecua et al. 2017; Arnott et al. 2021; Daykin et al. 2021), lower crime rates, and higher levels of life satisfaction level amongst the residents of a community. Furthermore, a study was conducted in the Netherlands to observe the effects of social capital on crime in the country. The results suggest that high levels of social capital provide an informal network of support for crime prevention as communities in the Netherlands with higher levels of social capital have relatively lower crime rates (Akçomak and ter Weel 2012a). Another study conducted in Japan also reveals similar results demonstrating the inverse relationship between social capital and crime (Takagi et al. 2012).

### Spatial social capital

Limited literature exists on the use of indicators/proxy variables or survey datasets to map geographical variations in social capital at a national scale. Sundquist et al. (2014) studied overall association between linking social capital and all-cause mortality in Sweden. They used neighbourhood voting participation rates as indicator of linking social capital mapped at small geographic units in Sweden (Sundquist et al. 2014).

Bennet Centre of Public Policy at the Cambridge University has recently published a blog in which they have explored social capital in the response to Covid-19. They mapped social capital at Local Authority level in UK using the number of community volunteer support groups that helped self-isolating and vulnerable people through the pandemic. They used this number (per local authority and population standardised) as a measure of social capital and its geographical variations to explore its correlations with socio-economic advantage, median age and average scores for wellbeing measures (Felici 2020).

In other studies survey derived points data and explanatory variables (environmental or demographic) have been used together in simulations to map social processes across geographical regions, see for example (Funkner et al. 2021; Ancona et al. 2022).

While surveys collect the opinion of respondents, their locational information (which is classed as personal) is not published along with the responses for survey data in order not to disclose their identity. Furthermore, national level surveys are summarised to provide statistical bulletins only.

### Aim and objectives

There is no national scale data available for Wales that can be directly used to measure varying level of social capital as explained above. To bridge this data and knowledge gap, this study aims to map social capital across Wales using secondary data sources. This is done by:

- Identifying key indicators of social capital that can be used to quantify social capital.
- Identifying and acquire secondary datasets that can be used as proxy variables for key indicators of social capital.
- Applying geospatial analysis to generate criterion maps (GIS layers) for the key indicators of social capital.
- Combining the key indicators together Using Analytical Hierarchy Process (AHP) to generate a social capital map for Wales.
- Validating the results using reflectors of high and low social capital.

## Methodology

### Identification of social capital key indicators

To measure social capital using the key indicators identified above, an investigation was carried out on existing datasets rather than conducting a new survey.

For bonding and bridging special capital, two national level surveys were identified which contained questions related to belongingness, trust, reciprocity, volunteer work, and view about local areas etc. There two surveys are: (i) British Household Panel Survey (BHPS Wave-18, 2009) and (ii) National Survey for Wales (NSW 2012–2013). The selected questions from these two surveys that are used in this research are provided in Online Annex-A. Both the BHPS and NSW datasets have been acquired at the Lower Super Output Areas (LSOA) using Special License Access. LSOA level is statically considered as the “small area” in terms of population (roughly 1500 per LSOA). The Special License Access provided us an LSOA number attached with each survey record that can be used to link it spatially and then be interpolated to map the survey results as explained in the sections below.

To measure civic participation, voter turnout data for 2012 local elections at ward level was used because it coincides with the two survey datasets used. However, local elections were not contested in all the wards in 2012, and therefore voting turnout from 2011 in the Welsh Assembly elections has been used for the wards where local elections were not contested in 2012. The two voter turnout datasets have been acquired from the Election Centre of Plymouth University (Plymouth-University 2013).

For mapping crime rates across Wales two datasets are used: (i) Rate of recorded criminal damage per 100 people of the daytime population (2008–2010) and (ii) the rate of adult offenders per 100 people of the daytime population (2008–2010). These indicators are acquired at the LSOA level from the Data Cymru Portal (InfobaseCymru 2010). For further validation of results, Welsh Index of Multiple Deprivation (WIMD) data have been used. The WIMD data Welis data were also acquired at the LSOA level from Welsh Government (WIMD 2011).

### GIS analysis and mapping of social capital indicators

Authors of this research, propose that Tobler’s first law of Geography can be the basis for mapping the indicators of social capital using the survey derived points data. Tobler’s law states: “All things are related, but nearby things are more related than distant things” (Tobler 1970). This law has formed the basis of many modern-day GIS analysis, particularly the interpolation techniques to fill the gaps between known data points.

To map the results of the selected questions from the two surveys, a spatial interpolation technique, i.e. Inverse Distance Weighting (IDW) was used. IDW is a two-dimensional interpolation function for irregularly-spaced data (Shepard 1968). A review of relevant literature suggests that spatial interpolation techniques have previously been used to successfully map survey responses: for example, public health variables at small scale were interpolated by (Meng et al. 2010). Inverse Distance Weighting (IDW) is used to spatially interpolate the averaged answer for each question.

**Fig. 1:**
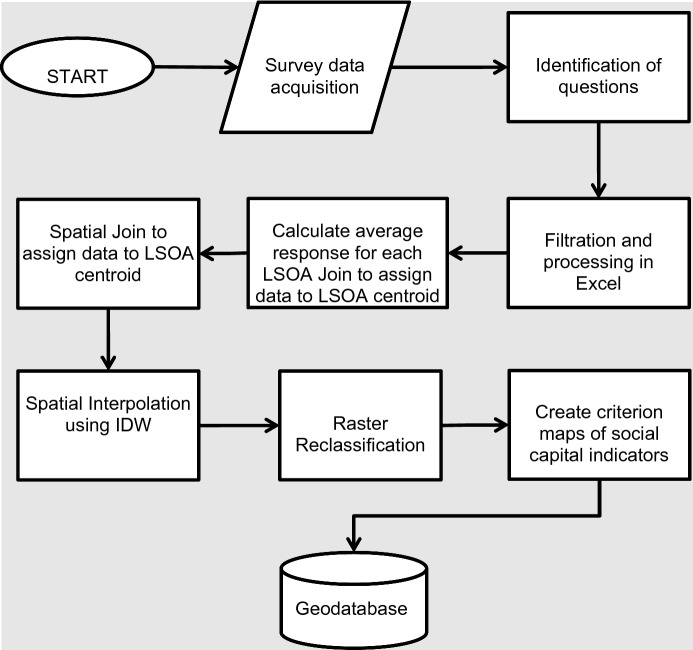
Processing and GIS analysis on survey data

IDW is an exact local interpolator, which means it does not cater for the global spatial autocorrelation into the consideration (Shepard 1968). It creates a surface that passes through the exact known values. Whereas the values at missing data points are interpolated based on its distance from and values at all the known data points, using this formula:

$$Z\left( x \right) = \mathop \sum \limits_{i = 0}^{n} \frac{wi(x)}{{\mathop \sum \nolimits_{i = 0}^{n} wi(x)}} zi$$

where *Z*(*x*) is the interpolated value at location *x*, *n* is total number of known data points, and *w*_*i*_(*x*) = $\frac{1}{{D(x, xi)^{2} }}$ which is the inverse distance weighting function (Shepard 1968). Figure 1 presents a flowchart showing the general process carried out to map the relevant survey data at the LSOA level in Wales. Records with no answers were removed for the selected questions, and average values (survey responses) are calculated for each LSOA. Some questions contained only binary answers such as ‘Yes’ and ‘No’ and some contained a range of numbers showing a preference scale, such as “best to worst”. An average value of the answer for each question is computed for each LSOA. Then, using the Spatial Join technique, these averaged values (of answers) are linked with the centroid of the corresponding LSOA. IDW interpolation is then applied as explained above.

Finally, raster reclassification was carried out to generate a GIS layer for individual social capital indicator, e.g. civic participation and social participation which are later used in the development of social capital map as explained in the sections below. This modelling was performed using the SEREN-SDSS (Irfan et al. 2017) to collate all the indicators together. The SEREN-SDSS uses a fishnet grid of 500 m^2^ in its geodatabase. Each criterion map is populated into this fishnet grid. The tool allows assigning relative weights to the indicators and sub-indicators within the AHP mode.

The following criterion maps have been developed using the statistical data and interpolated responses of the selected questions from the two surveys.

#### Civic participation

For analysing civic participation across Wales, voter turnout data for 2012 local elections and Welsh Assembly elections 2012 have been used at the ward level. These voter turnout datasets have been acquired from the Election Centre of Plymouth University (Plymouth-University 2013).To create a GIS layer for civic participation, Spatial Join tool in ArcGIS software was used. In this way each cell in the given column (representing the civic participation layer) of the fishnet gird is populated by the voter turnout information of the nearest electoral ward.

**Fig. 2:**
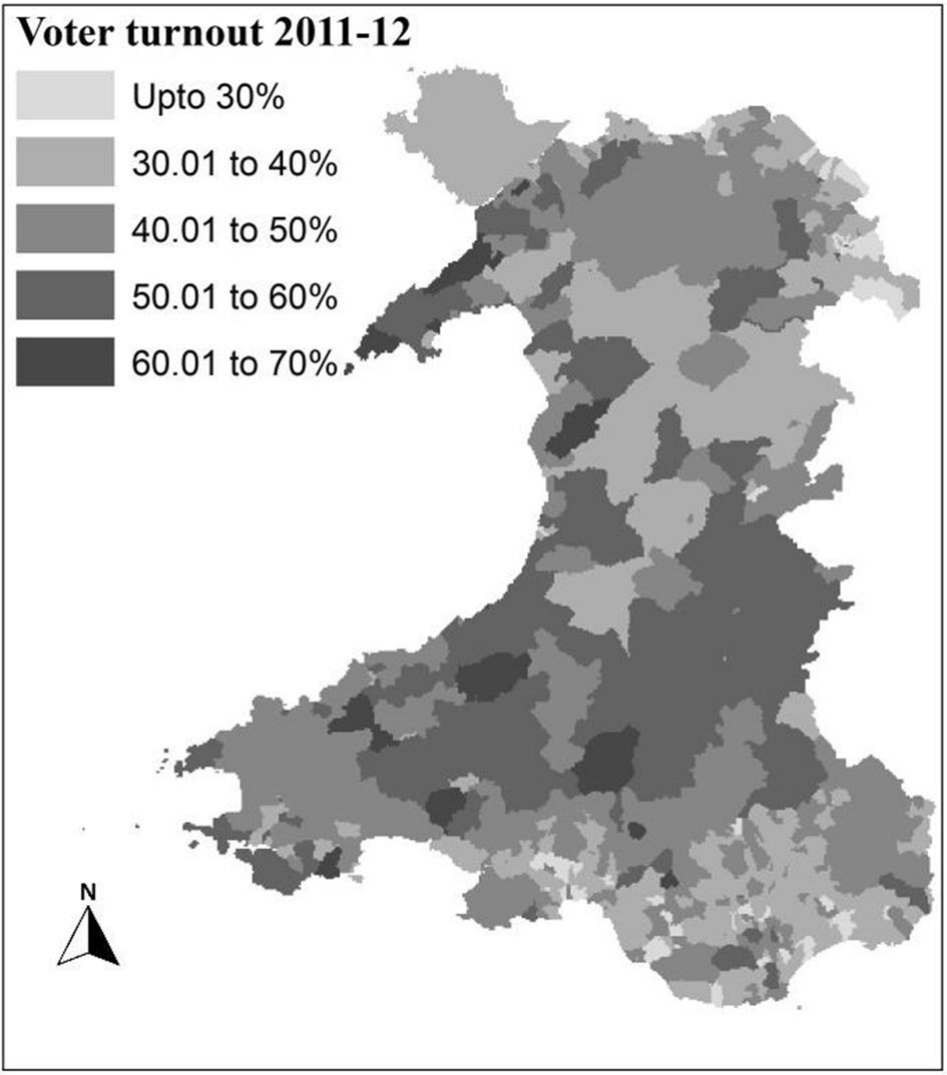
Civic participation—voter turnout from 2011 and 2012 elections

As shown in Fig. 2, civic participation (as indicated by voter turnout) is relatively higher in parts of mid, northwest, and west Wales. South Wales, which includes large towns such as Cardiff, Newport, and Swansea, seems to have lower levels of civic participation.

#### Social participation

There are no direct questions related to the “social participation” in NSW data. For this reason, the BHPS survey Wave-18 (2009) is selected. This data was acquired with a special licence. Three questions that could be used as sub-indicators to map “social participation” were identified. These questions are:

- Attends religious services
- Attends local group/voluntary organisation
- Do unpaid voluntary work

There are 1417 respondents from Wales in the dataset. 1289 records out of 1417 had a response to all three of these questions covering 598 LSOAs out of 1896. The results are scaled from 1 to 5, where 1 refers to “Very Frequently” and 5 to “Never”.

**Fig. 3:**
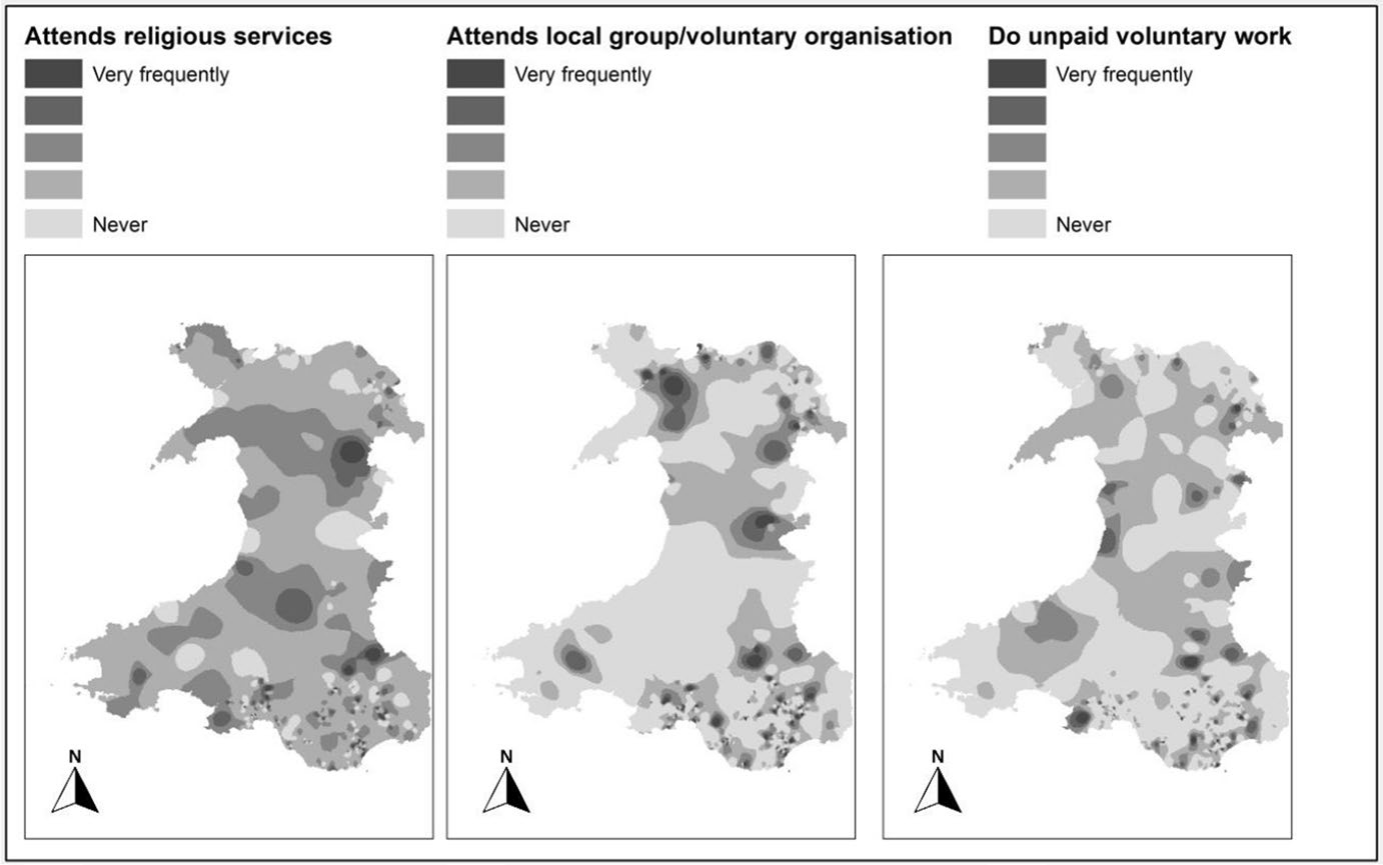
Sub-indicator maps of “Social participation”

Social participation maps produced using this data (Fig. 3) don’t show a clear trend; however, these are key indicators to be used in the analysis and development of the overall social capital map of Wales.

#### Views of the local area

The questions about the views of the local area were taken from the NSW dataset, which has more records compared to BHPS. Since it is Wales only survey, it provides an opportunity for more detailed mapping. The following three questions are identified from the NSW as sub-indicators of “views of the local area”:

- Belonging to the neighbourhood: There are 14,552 records in the dataset. The same approach is used as described in the previous section to map the survey results of this question across Wales. 14,481 records out of 14,552 had a valid response to this question covering 1881 LSOAs out of 1896. The result is scaled on 1 to 5 where 1 refers to “Strongly Agree” and 5 to “Strongly Disagree”.
- Safety at home after work: The same procedure is applied to map the survey results of this question across Wales. 14,453 records out of the 14,552 valid responses to this question covering 1881 LSOAs out of 1896 were extracted. The result is scaled on 1 to 4 where 1 refers to “Very Safe” and 4 to “Very Unsafe”.
- Safety walking in the local area after dark: As above, the same procedure applied to map the survey results of this question across Wales. 14,428 records out of 14,552 had responded to this question covering 1879 LSOAs out of 1896. The result is scaled on 1 to 4 where 1 refers to “Very Safe” and 4 to “Very Unsafe”.

**Fig. 4:**
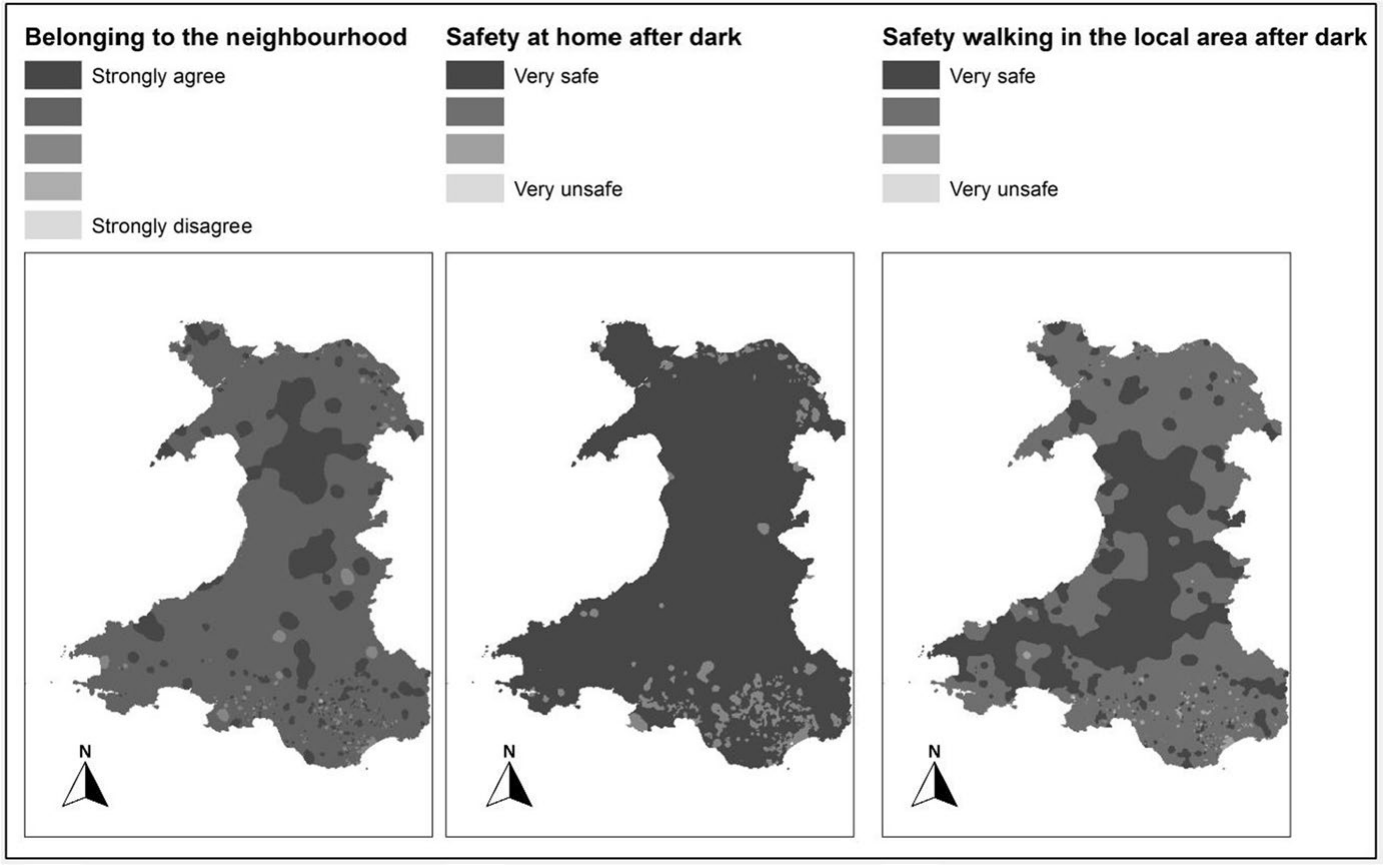
Maps of view about the local area

These maps representing the views of the local area (Fig. 4) show some trends with respect to urban rural divide, i.e. people in semi urban and rural areas feeling safe at home, feeling safe walking in their local areas, and feeling more belongingness to their neighbourhoods as compared to urban and densely populated areas in South Wales.

#### Reciprocity and trust

To map this indicator of social capital, NSW (National Survey of Wales) 2012–2013 data is acquired from Welsh Government with a special license to have LSOA linkage. NSW has more records as compared to BHPS and is specifically designed for Wales only and therefore provides an opportunity for more detailed mapping. The following four questions were identified from the NSW as useful sub-indicators of “reciprocity and trust”:

- Trusting people in the neighbourhood: The same procedure outlined above is applied to map the responses to all the following selected questions across Wales. 13,974 records out of 14,552 had response to this question covering 1878 LSOAs out of 1896. Records with “just moved to the area” are also removed. The result is scaled from 1 to 4 where 1 refers to “Many people in the neighbourhood can be trusted” and 4 to “None of the people in the neighbourhood can be trusted”.
- Safe for children to play outside: 14,334 records out of 14,552 had response to this question covering 1881 LSOAs out of 1896. The result is scaled on 1 to 5 where 1 refers to “Strongly agree” and 5 to “Strongly disagree”.
- People from different background get on well together: 13,368 records out of 14,552 had response to this question covering 1881 LSOAs out of 1876. The result is scaled from 1 to 5 where 1 refers to “Strongly agree” and 5 to “Strongly disagree”.
- People treating each other with respect and consideration: 14,442 records out of 14,552 had response to this question covering 1880 LSOAs out of 1876. The result is scaled from 1 to 5 where 1 refers to “Strongly agree” and 5 to “Strongly disagree”.

**Fig. 5:**
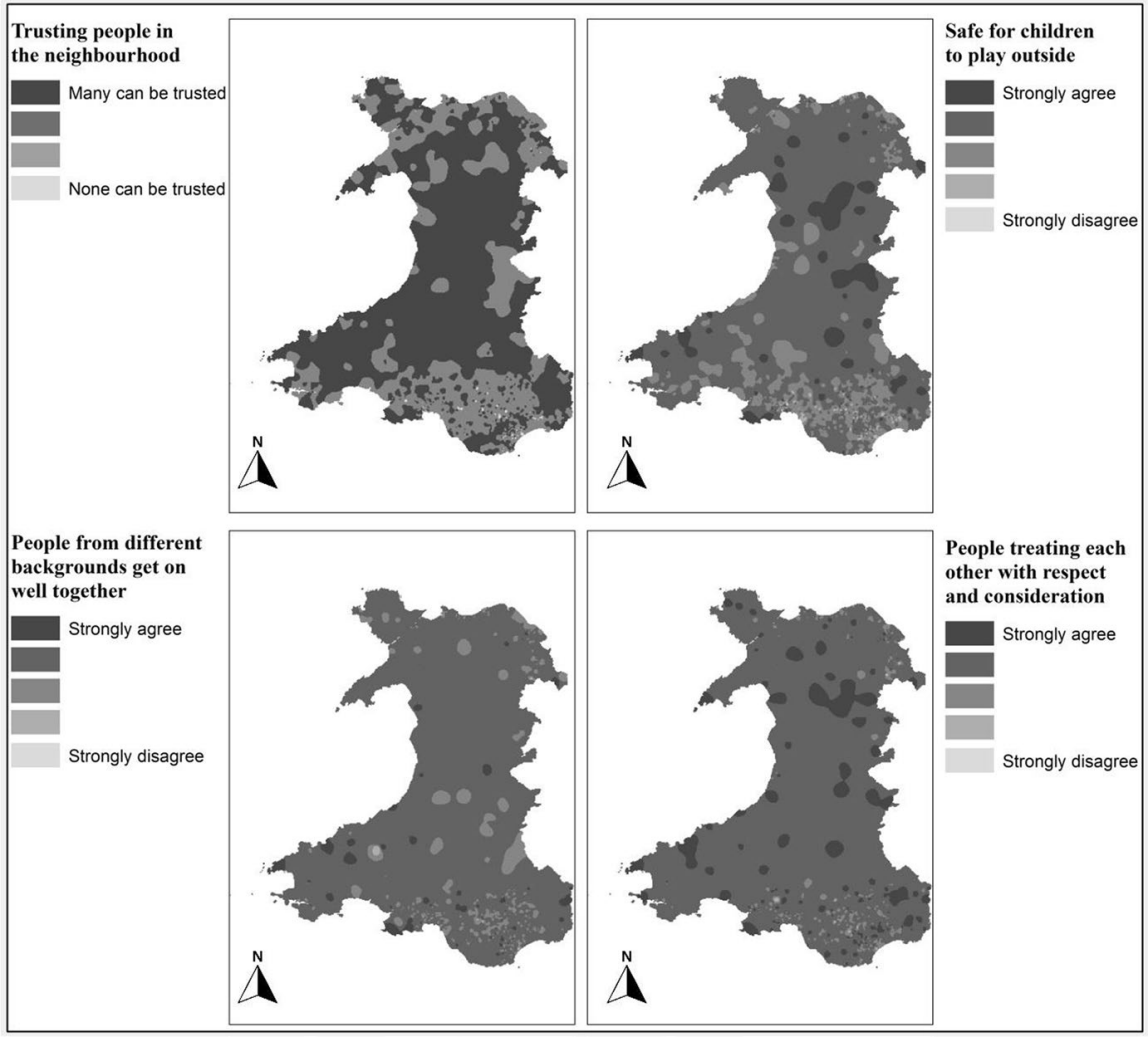
Indicator maps of “Reciprocity and trust”

The maps representing the reciprocity and trust (Fig. 5) show some trends: for example, people in mid and west of Wales shows higher trust in their neighbourhood. However, it is only significant when combined with other indicators for the analysis and development of the overall social capital map of Wales.

#### Crime rates

As explained in “Identification of social capital key indicators” section, two datasets are used for mapping Crime across wales, i.e. (i) Rate of recorded criminal damage per 100 people of the daytime population (2008–2010) and (ii) the rate of adult offenders per 100 people of the daytime population (2008–2010). These indicators are acquired at the LSOA level from (InfobaseCymru 2010) and assigned to Fishnet cells.

To create a GIS layer for crime rates, Spatial Join tool in ArcGIS software was used. In this way each cell in the given columns (representing the rate of recorded criminal damage and rate of adult offenders) of the fishnet gird is populated by the crime rate information of the nearest LSOA.

**Fig. 6:**
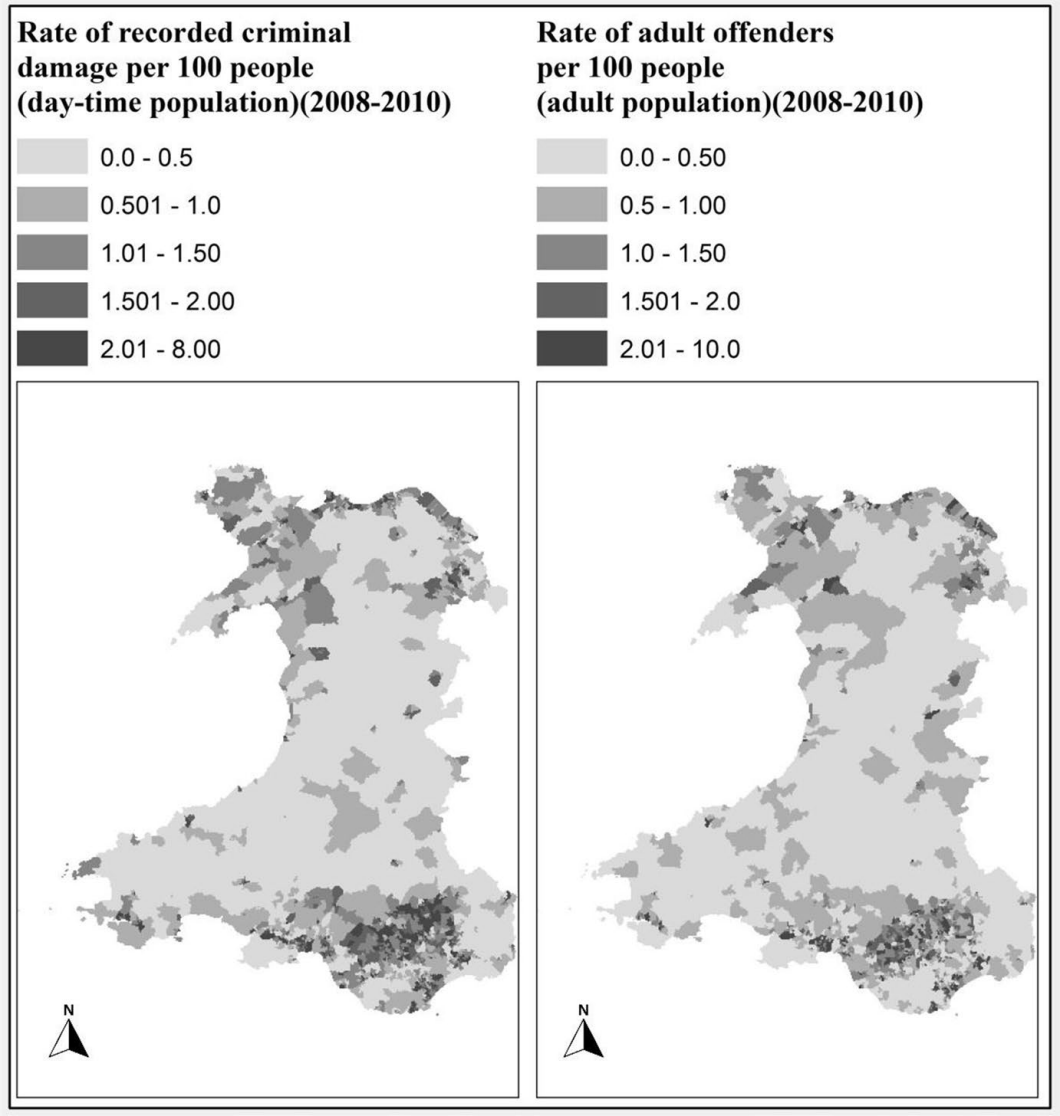
Indicator maps of “Crime rate”

The maps representing the crime rates (Fig. 6) show some trends: for example, higher crime rates in South Wales compared to mid and west Wales. However, it is only significant when combined with other indicators for the analysis and development of the overall social capital map of Wales. This data will also be used further to validate the results as explained in “Validation of results” section.

### Analytical hierarchy process (AHP)

As explained in “Introduction” section, several indicators or proxy variables have been used by others as a measure of social capital types. Since we wanted to map a general level of social capital which covers both objective and subjective components, it required an empirical way to combine these together. In the absence of any recognised empirical formula to combine these components, we used AHP technique for this purpose by creating a hierarchical structure of indicators and sub-indicators. This was performed using the AHP tool of the SEREN-SDSS (Irfan et al. 2017) to collate all the indicators together to map social capital across Wales. The SEREN-SDSS uses a fishnet grid of 500 m^2^ in its geodatabase. Each criterion map is populated into this fishnet grid. The tool allows assigning relative weights to indicators and sub-indicators within the AHP model.

**Fig. 7:**
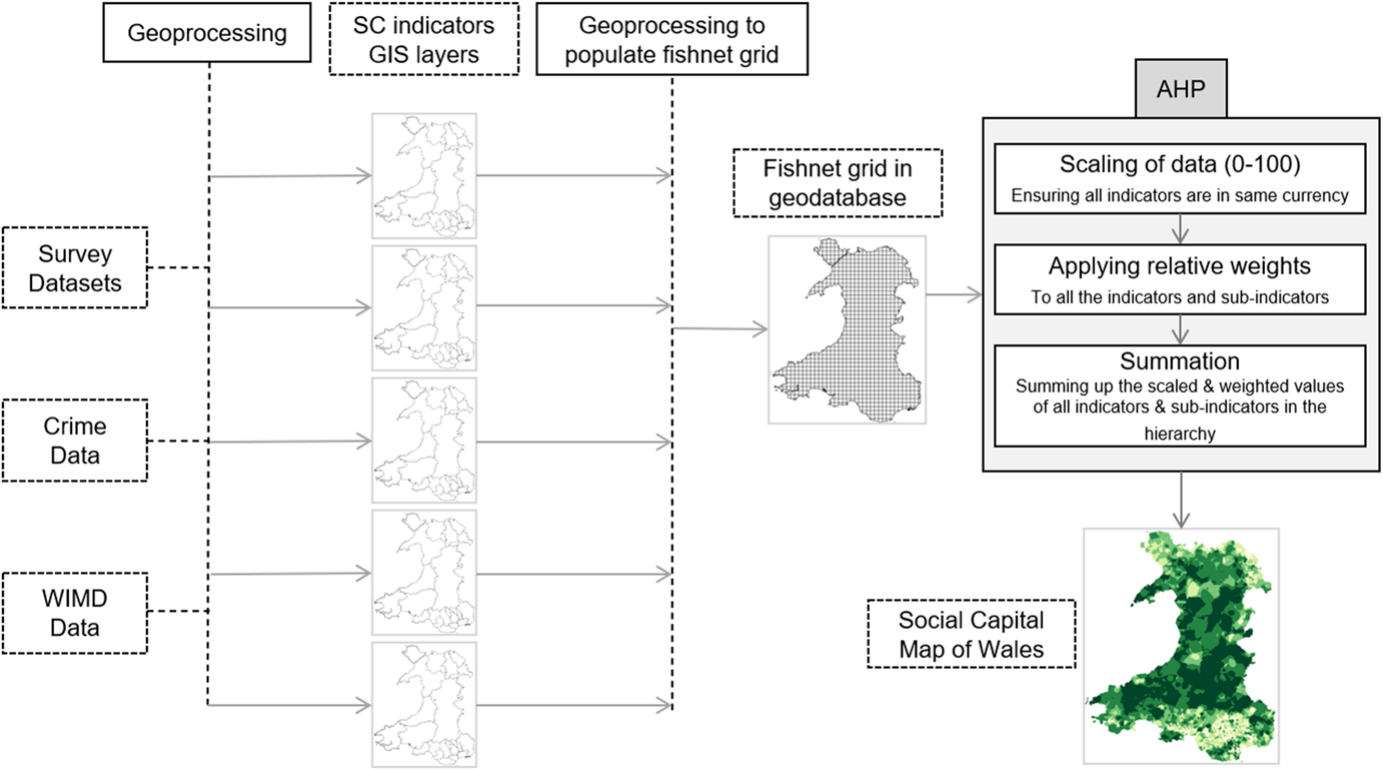
Overall process of developing social capital map of Wales using data, geoprocessing and AHP summation process

In this model, an equal weight has been assigned to all the indicators. All the main indicators (i.e. “crime rate”, “social participation”, “views of the local area” and “reciprocity and trust”) are “benefit” (i.e. a positive or direct relation with social capital) in nature, which means the higher their values are, the more they contribute to a higher rating of social capital. All the base indicators (i.e. the survey question response and crime rates) are “cost” in nature (negative correlation), which means the lower their values are, the more they contribute to a higher rating of social capital. In both surveys (NSW and BHSP), responses are organised in a way that a value of 1 represents the most satisfied. For example, the BHSP question on attending religious services has possible answers from 1 to 5, where 1 represents ‘Very frequently’ and 5 represents ‘Never’, which is why it is treated as a ‘Cost’ indicator. More details on survey questions used in the analysis are provided in Online Annex-A. Figure 7 below summarise the entire process of developing the social capital map for Wales. It follows these steps:

- Starts with raw data (surveys, crime rates etc.) selected to represent key indicators.
- Geoprocessing is then applied to the data to generate criterion maps (individual GIS layers for each indicator or sub-indicators). Type of geoprocessing used is based on the type of data as explained in "GIS analysis and mapping of social capital indicators" section.
- Fishnet is then populated with each criterion map. There is an individual column in the grid for each indicator and sub-indicators. The fishnet approach was taken because each criterion map level of detail could be different, for example some data was available at the LSOA level, while another was at electoral Ward level or produced by interpolating the survey responses. Fishnet technique allowed all this data to be available at the same geography level (i.e. the fishnet grid cell). This step was necessary to apply AHP summation process. Once fishnet is fully populated it was added to the geodatabase of the AHP tool of the SEREN-SDSS.
- Scaling was carried out to bring each sub-indicators and indicators in the fishnet to bring all the data in the same currency. This was carried out because AHP summation requires each data to be in the same units. Without scaling, the summed-up number will not make any sense. For our case, we scaled everything between 0 and 100, where the value 0 means no contribution towards the calculation of social capital and 100 means highest contribution towards the calculation of the social capital value.
- Relative weights are then applied to each indicator and sub-indicators. In our case equal weights were assigned, although the AHP tool of SEREN-SDSS allows assigning different relative weights to indicators/ sub-indicators in the AHP hierarchy if you want to put more weight behind any component within the hierarchy.
- Finally, AHP summation process was applied to generate the overall social capital map. Sub-indicators (scaled and weighted) summed-up together to calculate the indicators they are feeding into the AHP hierarchy. Then all indicators (scaled and weighted) summed-up to calculate the overall social capital measure for each cell in the fishnet grid.

**Fig. 8:**
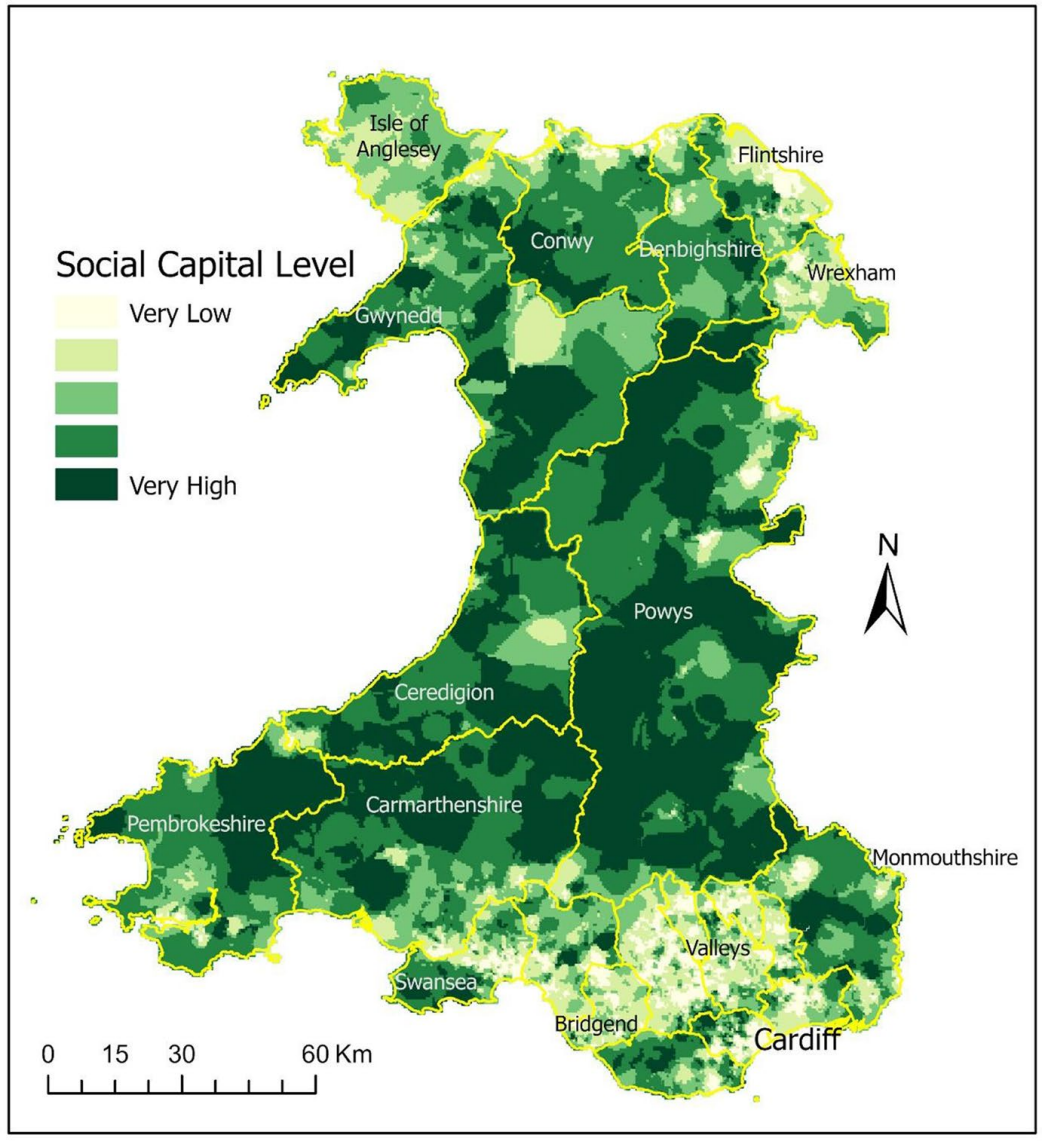
Social capital map of Wales—using equal weights for all indicators in the AHP

The resultant social capital map of Wales is shown in Fig. 8, along with indicators’ relative weights used in the AHP process. AHP is a multicriteria decision analysis technique which can be used in such scenarios where an empirical context is not present. Although we applied equal weights to all the indicators used in the AHP hierarchy, one can argue and change the relative weights as required. For example, if the evidence suggests that bridging capital is more important than bonding capital for a particular context, we can simply assign more weight to it. So much so, 100% weight can be assigned to it to represent only the bridging social capital. The final map brings together everything in the same currency where a higher number shows a higher value of the particular feature being mapped, in this case social capital.

Final social capital map of Wales shown in Fig. 8 shows some trends of relatively higher social capital in North and West of Wales compared to the South Wales. Similarly, there is a noticeable trend of relatively higher social capital in the rural areas compared to the densely populated urban areas in Southeast and Northeast of Wales.

## Validation of results

As explained in “Introduction” section, the social capital has many dimensions. Some are objective such as the observable and tangible networks, associations, and institutions in a society, also the measures such as the civic participation and social participation. Whereas, the subjective aspects relate to issues such as mutual trust, reciprocity, and generally accepted attitudes, norms, and behaviours (Putnam 1993; Grootaert and Van Bastelaer 2002; Foxton and Jones 2011).

In this research, we aspired to combined as many of these components together, to quantify and map a general level of social capital across Wales. In the absence of any recognised empirical formula to combine these components, we used AHP technique for this purpose by creating a hierarchical structure of indicators and sub-indicators as explained in “Methodology” section.

To validate the social capital map produced using this methodology, we carried out a series of geostatistical analysis to test the correlation between produced social capital map with crime and multiple deprivation data as explained in the sections below:

### Social capital validation using crime data

GIS analysis was performed to identify any trends between crime data and the social capital map produced. For this purpose, the AHP-based site selection tool of the SEREN-SDSS (Irfan et al. 2017) was used. First, the crime data was selected-out of the AHP so that it does not contribute to the mapping of social capital. The AHP is then applied across Wales using the rest of the indicators with an equal relative weight to produce the social capital map. The top and bottom 20% of cells (with respect to the social capital and crime data) were then exported as separate layers. There are 17,372 cells in each of the four layers (20% of 86,860). The “Intersection tool” in ArcGIS was then used to select only the following areas:

- Top 20% cells of social capital intersecting with the top 20% cells of crime.
- Bottom 20% cells of social capital intersecting with the bottom 20% cells of crime.
- Top 20% cells of social capital intersecting with the bottom 20% cells of crime.
- Bottom 20% cells of social capital intersecting with the top 20% cells of crime.

**Fig. 9:**
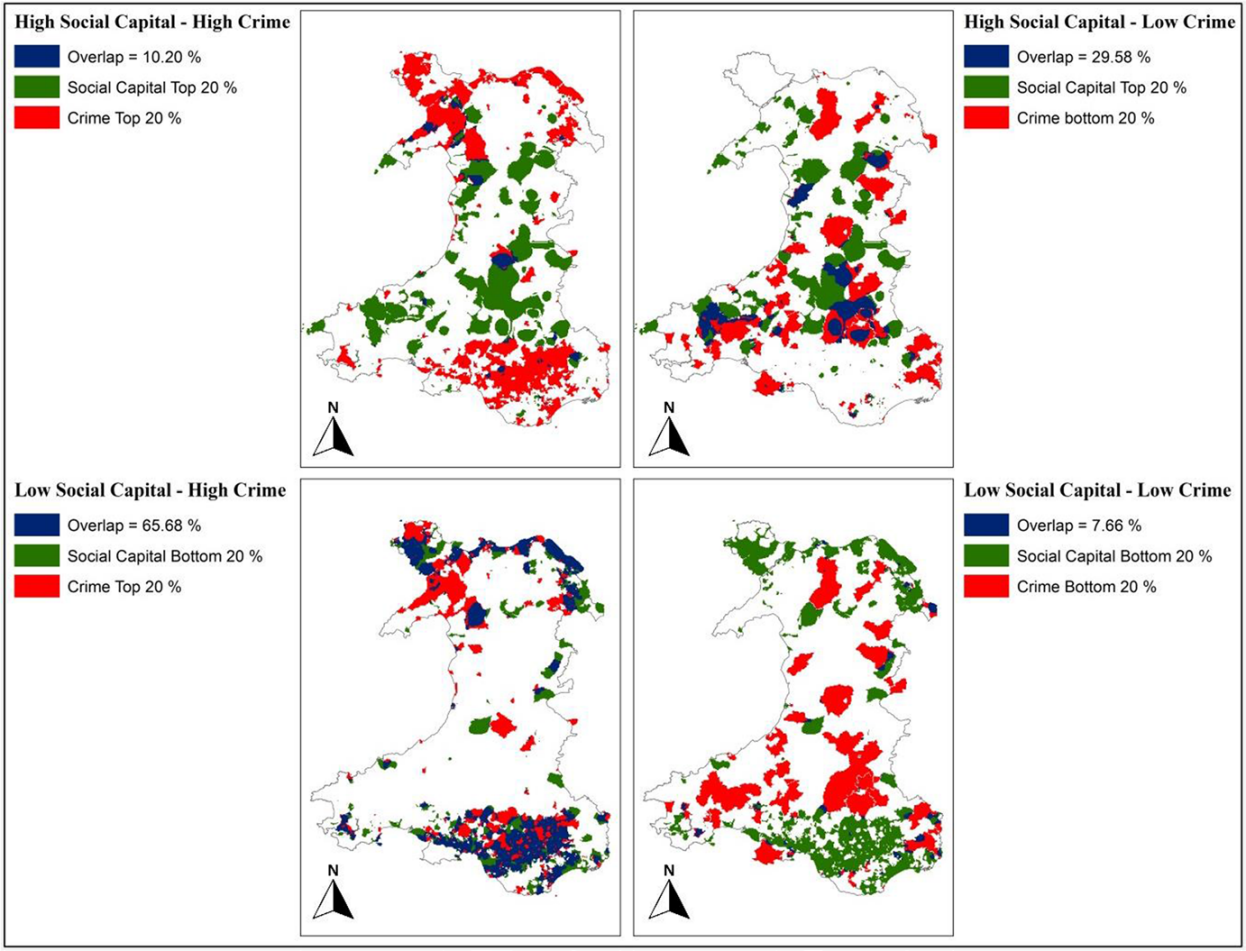
Social capital results verification using crime rates

The results are shown in Fig. 9, where the two intersecting layers are given for each analysis. The number or intersected cells (out of 17,372) are shown along with the percentage of cells being intersected. Validation was carried by analysing whether the areas of higher social capital coincide with low crime rates, and the areas with low social capital coincide with the higher crime rates.

Validation results are presents in Fig. 9. Promisingly, the results show that the areas with low social capital co-locate with high crime rates (i.e. 65.68%). Similarly, areas with high social capital co-locate with low crime rates. These findings are very similar to those of (Akçomak and ter Weel 2012a) and (Takagi et al. 2012) discussed previously.

Since crime rates themselves are a key indicator of social capital, we suggest that the method used here to map social capital across Wales is potentially robust. With the inclusion of crime rate data (after validation of social capital map generated without using it as an indicator) back into the model, the generated social capital map can be used with more confidence.

**Fig. 10:**
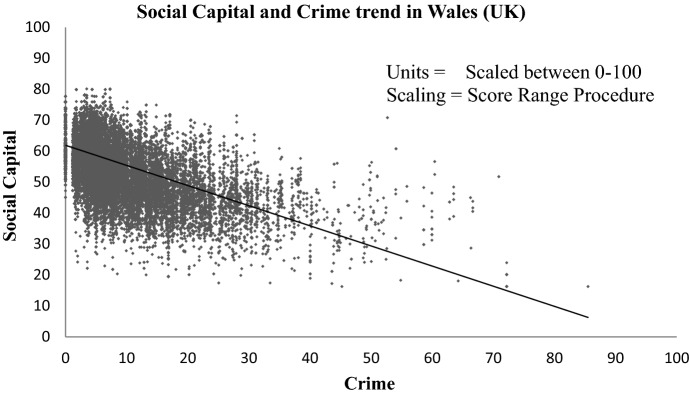
Social capital (mapped without using Crime indicators) results verification using crime rates showing a trend (linear fit) between higher social capital and lower crime rate

Figure 10 shows the trend between Social Capital and Crime in Wales. There is a clear negative trend found between the two, i.e. areas with higher social capital tend to have lower rates of crime.

### Social capital and multiple deprivation

As a second validation process, the Welsh Index of Multiple Deprivation (WIMD) has been used. In Wales, the WIMD is an official means of ranking areas in terms of multidimensional deprivation. In WIMD 2011 dataset, Wales is divided into 1896 Lower Super Output Areas (LSOA). Rank-1 is rated the most deprived LSOA in Wales, whereas 1896 is the least deprived LSOA.

**Fig. 11:**
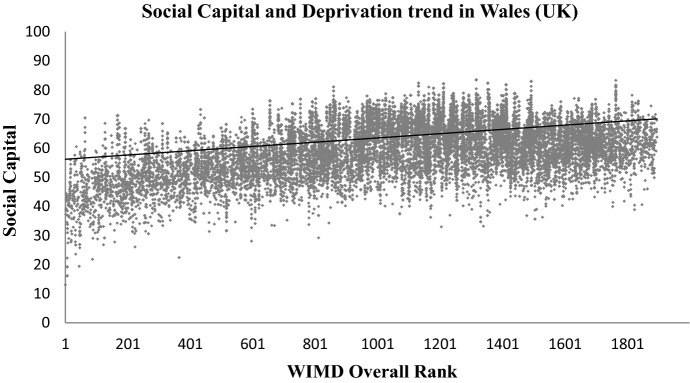
Social capital’s trend (linear fit) with overall multiple deprivation in Wales. Low WIMD rank means more deprived and a higher rank means less deprived

**Fig. 12:**
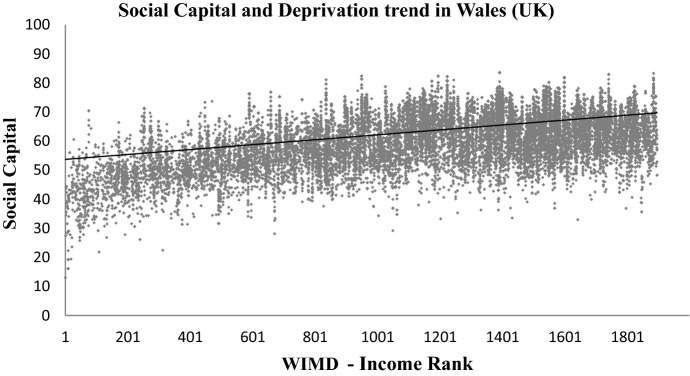
Social capital’s trend (linear fit) with income deprivation in Wales. Low WIMD rank means more deprived and a higher rank means less deprived

**Fig. 13:**
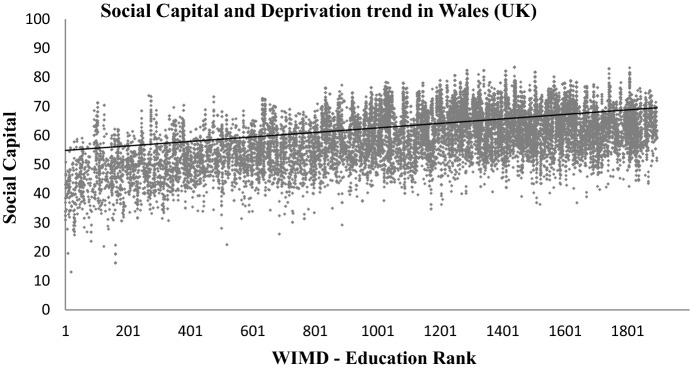
Social capital’s trend (linear fit) with education deprivation in Wales. Low WIMD rank means more deprived and a higher rank means less deprived

WIMD is constructed from eight different domains of deprivation, i.e. income, housing, employment, access to services, education, health, community safety, physical environment, and an overall index (WIMD 2011). These WIMD indexes are also acquired at the LSOA level from Data Cymru portal (InfobaseCymru 2010). As highlighted in the literature review, higher social capital is linked with higher education and higher income levels of the community, see for example (Putnam 1993; Anderson et al. 2012; Liu et al. 2014; Fernández Aldecua et al. 2017; Daykin et al. 2021). To further validate the method of mapping social capital in Wales proposed in this paper, the resultant social capital map has been plotted to explore the trend with (i) the WIMD overall index, (ii) the WIMD Education Index, and (iii) the WIMD Income index. The three indexes have been selected due to the potential links of social capital with education, income levels and deprivations of communities as explained in the literature review. The results are shown in Figs. 11, 12, 13.

Figures 11, 12, 13 show a visible trend between social capital and the three indices of deprivation from the WIMD. Areas with higher social capital are mostly those where education, income and overall (or multiple) deprivation is low. Areas with relatively lower social capital are those where education, income and overall (or multiple) deprivation is high. Note that Rank-1 in WIMD datasets is described as the most deprived area (LSOA) in the entire country.

Results of our validation are also consistent with those presented in the social capital analysis in Scotland, where social capital measures in the 20% most deprived areas were compared with the least deprived areas (Kerri McClymont 2020). In Scotland, although most deprived and least deprived areas have similar levels of social interaction but people in the least deprived areas have been observed to experience loneliness, and have lower neighbourhood ratings (Kerri McClymont 2020).

## Conclusions

Paper first shows evidence from the literature that social capital has proven to be a useful consideration in tackling societal and environmental challenges in different parts of the world. Like many other regions, Wales is also going through a big societal change to reach Net Zero by 2050. We believe that social capital can play a vital role in this regard since behaviour change in our society will be key to success. Also, the fact that decarbonisation will rely heavily on smart local energy systems since everything cannot be done at the UK level. Increased uptake of local renewable energy systems and their sustainable success is linked with how people and communities effectively work together to adopt this big shift. This is where social capital comes into play.

However, we have identified a knowledge and data gap in this regard since there is no dataset available which shows level of social capital in different parts of Wales. To fill this gap, we envisaged that existing secondary datasets can potentially be used to measure and map a general level of social capital across the country.

After recognising the data gap, we carried out a literature review and identified the key indicators and proxy variables that can be used to map a relative general level of social capital across the country rather than conducting a new survey. We then identified and acquired existing datasets that can be used as proxy variables for this purpose. We identified questions found within two existing national level surveys that can be used to measure bridging and bonding social capital. The survey responses were acquired from the Welsh Government through a special access licence for this research because we required the location information of the survey respondents, for us to use it in GIS analysis. The locational information of the survey respondents was given in the form of LSOA number, the respondents belonged to. IDW technique was used in GIS system to spatially interpolate the survey responses from the centroids of LSOAs. Other dataset used as a proxy variable of social capital is the voter turnout data at ward level and the crime statistics at the LSOA level. All these indicators were then populated into a fishnet grid within the GIS system. Since there is no standard empirical formulation that could be used to combine all these indicators together to calculate overall level of social capital. Therefore, a multicriteria decision analysis technique was used in the form of Analytical Hierarchy Process by assigning equal weights to combine all the indicators together in the form of GIS map showing relative general level of social capital across the country.

To validate our results, we used some other datasets which are known (through literature review) to have strong correlation (negative or positive) with social capital, e.g. crime rates, income, education, and overall deprivation. Our validations results demonstrated these correlations, e.g. areas of high income and high education attainments, collocate with areas of higher social capital. Similarly, areas of higher crime rates, collocate with areas of lower social capital. Note that the crime rates data was removed from the AHP process before this validation was carried out. The satisfactory results of the validation process give us the confidence that social capital maps produced in this way, using secondary datasets, geoprocessing and AHP technique can be reliably used by policy makers in national and local governments.

## Limitations and future research

This paper does not explicitly test the assumed link between social capital and accelerating decarbonisation. We intend to use this data for further research in exploring the link between social capital and attitudes and receptiveness towards decarbonisation interventions. For this purpose, we are currently undertaking a study in a local authority (Caerphilly) in South Wales. We have conducted a short citizen survey that will help us establish the attitude and receptiveness towards decarbonisation in different parts of the local authority. The survey will help us ascertaining what participants think about climate change; their willingness to contribute towards reducing their carbon footprint; and what help from their local authority will be most beneficial in this regard. We will analyse and identify any geographical trends in these responses and then explore what correlation (if any) it shows with varying levels of social capital in the local authority.

Furthermore, if we can identify any meaningful trends, we plan to carry out further research into designing place-based decarbonisation policies and strategies which are suited to the prevailing level of social capital in different parts of the country. Similarly, what can be done to improve the social capital in certain areas where it is relatively low, so that communities living in those areas, don’t miss out on the co-benefits (e.g. local green jobs, business growth, lower energy bills etc.) of decarbonisation interventions through public and private investments.

**Table:** 

Data	Provider	Geographical identifier	Availability
British Household Panel Survey Wave-18 (2009)	(UK DATA SERVICE, UK DATA ARCHIVE, UNIVERSITY OF ESSEX, WIVENHOE PARK, COLCHESTER, ESSEX, CO4 3SQ, UK)	Lower Super Output Area (LSOA) level	Not publicly available. Acquired through special license (due to the associated Lower-level Geographical Identifiers)
National Survey of Wales 2012–2013	Knowledge and Analytical Services Welsh Government Cathays Park Cardiff CF10 3NQ	LSOA level	Not publicly available. Acquired through special license (due to the associated Lower-level Geographical Identifiers)
Welsh Index of Multiple Deprivation 2011	Local Government Data Unit Wales via their InfoBaseCymru data portal https://www.infobasecymru.net/IAS/	LSOA level	Publicly available
Crime Rates (recorded criminal damage and the rate of adult offenders per 100 people of the daytime population (2008–2010)	Local Government Data Unit Wales via their InfoBaseCymru data portal https://www.infobasecymru.net/IAS/	LSOA level	Publicly available
Voter turnout data at electoral ward level (2012 local elections at ward level and the Welsh Assembly elections 2012	Election Centre of Plymouth University	Electoral wards	Not publicly available. Acquired through special license

## Electronic supplementary material

Below is the link to the electronic supplementary material.

Supplementary file1 (DOCX 39 kb)

## Data Availability

Manuscript contains results of the analysis of two national survey datasets (British Household Panel Survey and British Household Panel Survey) and an electoral voter turnout dataset at a geographical scale, which is not publicly available. This data was made available to the first author through a special access licence for doctoral research. Other datasets used in the analysis are publicly available through StatsWales and InfoBaseCymru data portals. Table below list the details of all the datasets used in this research.
