# Supplementary material for: Mapping social capital across Wales (UK) using secondary data and spatial analysis
Source: SN Soc Sci. 2023 Mar 6;3(3):56. doi: 10.1007/s43545-023-00639-1 (PMC9987399; doi:10.1007/s43545-023-00639-1)
Supplement: Supplementary file 1 — Supplementary file1 (DOCX 39 kb) [file 43545_2023_639_MOESM1_ESM.docx]

**Annex-A**

| **Question** | | **Answer Type** | **Total respondents** | | **Total LSOAs represented** | **Potential indicators** |  |
| --- | --- | --- | --- | --- | --- | --- | --- |
| **British Household Panel Survey Wave-18 (2009)** | | | | | | | |
| Likes present neighborhood | | Yes 1  No 2 | | 1402 | 672 | Views of the local area |  |
| Attends religious services  Classes modified as: 4 into 5 (Never)  5 into 4 (for weddings etc.) | | 1 Very frequently  5 Never | | 1289 | 597 | Social Participation |  |
| Attend local group/voluntary organisation | | 1 very frequent  5 Never | | 1289 | 597 | Social Participation |  |
| Do unpaid voluntary work | | 1 very frequent  5 Never | | 1289 | 598 | Social Participation |  |
| **National Survey of Wales 2012-2013** | | | | | | | |
| Belonging to local area? | 1 Strongly Agree  5 Strongly Disagree | | | 14481 | 1881 | Views of the local area |  |
| People like to help their neighbours? | 1 Strongly Agree  5 Strongly Disagree | | | 14414 | 1880 | Social network & Social Support |  |
| Safety at home after dark? | 1 Very Safe 4 Very Unsafe | | | 14537 | 1881 | Views of the local area |  |
| Safety walking in local area after dark? | 1 Very Safe 4 Very Unsafe | | | 14287 | 1879 | Views of the local area |  |
| Trusting people in the neighbourhood?  (records with “5 just moved in the area” also removed) | 1 Many people in the neighbourhood can be trusted  4 None of the people in the neighborhood can be trusted | | | 13974 | 1878 | Reciprocity and Trust |  |
| Safe for children to play outside? | 1 Strongly Agree  5 Strongly Disagree | | | 14334 | 1881 | Reciprocity and Trust |  |
| People from different backgrounds get on well together  (records with “6 too few people in the area” removed)  (records with “7 all same background” removed) | 1 Strongly Agree  5 Strongly Disagree | | | 13368 | 1876 | Reciprocity and Trust |  |
| People treating each other with respect and consideration | 1 Strongly Agree  5 Strongly Disagree | | | 14442 | 1880 | Reciprocity and Trust |  |
